# Minute ventilation of cyclists, car and bus passengers: an experimental study

**DOI:** 10.1186/1476-069X-8-48

**Published:** 2009-10-27

**Authors:** Moniek Zuurbier, Gerard Hoek, Peter van den Hazel, Bert Brunekreef

**Affiliations:** 1 Public Health Services Gelderland Midden, Arnhem, The Netherlands; 2 Institute for Risk Assessment Sciences (IRAS), Division of Environmental Epidemiology, Utrecht University, Utrecht, The Netherlands; 3 Julius Center for Health Sciences and Primary Care, Utrecht University, Utrecht, The Netherlands

## Abstract

**Background:**

Differences in minute ventilation between cyclists, pedestrians and other commuters influence inhaled doses of air pollution. This study estimates minute ventilation of cyclists, car and bus passengers, as part of a study on health effects of commuters' exposure to air pollutants.

**Methods:**

Thirty-four participants performed a submaximal test on a bicycle ergometer, during which heart rate and minute ventilation were measured simultaneously at increasing cycling intensity. Individual regression equations were calculated between heart rate and the natural log of minute ventilation. Heart rates were recorded during 280 two hour trips by bicycle, bus and car and were calculated into minute ventilation levels using the individual regression coefficients.

**Results:**

Minute ventilation during bicycle rides were on average 2.1 times higher than in the car (individual range from 1.3 to 5.3) and 2.0 times higher than in the bus (individual range from 1.3 to 5.1). The ratio of minute ventilation of cycling compared to travelling by bus or car was higher in women than in men. Substantial differences in regression equations were found between individuals. The use of individual regression equations instead of average regression equations resulted in substantially better predictions of individual minute ventilations.

**Conclusion:**

The comparability of the gender-specific overall regression equations linking heart rate and minute ventilation with one previous American study, supports that for studies on the group level overall equations can be used. For estimating individual doses, the use of individual regression coefficients provides more precise data. Minute ventilation levels of cyclists are on average two times higher than of bus and car passengers, consistent with the ratio found in one small previous study of young adults. The study illustrates the importance of inclusion of minute ventilation data in comparing air pollution doses between different modes of transport.

## Background

Recently, there is an increasing number of studies on exposure to air pollution in different modes of transport, i.e. train, car, bus, bicycle and by foot [1-9]. The in-vehicle air pollution levels have generally been found to be slightly higher than exposure levels of cyclists and pedestrians [3]. Only few studies have taken into account that pedestrians and cyclists have an increased minute ventilation compared to other commuters, influencing their inhaled dose of air pollutants. To correct for the higher minute ventilation of cyclists, Van Wijnen and Rank [4,5] use a factor obtained by Vrijkotte [10]. Vrijkotte measured minute ventilation in nine young adults (mean age 25, four women, five men), during 20 minutes of cycling at their personally preferred speed, and during 10 minutes while driving a car. Only mouth inhalation was measured, persons were wearing a nose clip to prevent nose respiration. Minute ventilation during cycling was 2.3 times higher than during car driving.

Minute ventilation is difficult to measure in field studies. However, it can be estimated by measuring heart rates during commuting. Heart rate is mainly influenced by oxygen consumption; the correlation between oxygen consumption and minute ventilation is high, thus heart rate and minute ventilation are expected to be strongly associated.

Samet et al [11,12] measured minute ventilation and heart rate in 15 healthy men and 15 healthy women during rest and while performing an exercise test on a bicycle ergometer, during which the test persons cycled at increasing intensity (heart rates between 80-140 beats per minute). Although heart rate predicted minute ventilation well, there was substantial variability in the quantitative relation between heart rate and minute ventilation between individuals.

The study Transport Related Air Pollution, Variance in commuting, Exposure and Lung function (TRAVEL) examines commuters' exposure to air pollution and related short term health effects. To study the relation between the health effects and air pollution, information was needed on the minute ventilation during commuting, to improve the estimation of the inhaled dose of air pollutants. Because of the limited information available on the relation between heart rate and minute ventilation and the unclear applicability of these data to other populations, we performed a bicycle ergometer study to examine the relation between heart rate and minute ventilation for all participants of the TRAVEL study. The aim of the bicycle ergometer study was to estimate minute ventilation levels of cyclists, car and bus passengers, and to study differences in estimations between using individual and average regression coefficients from this and previous studies. Calculated relations between heart rate and minute ventilation are applied to two hour heart rate recordings, to esteem differences in minute ventilation levels during commuting by car, bus and bicycle.

## Methods

### TRAVEL study

The TRAVEL study examines exposure to air pollution of cyclists, car passengers and bus passengers. In addition, short term effects of these exposures are examined on lung function, air way resistance, exhaled nitrogen oxide levels and blood markers, among others markers of inflammation and coagulation. Volunteers were recruited through intranet websites of their employer. All volunteers were civil servants working in Arnhem, the Netherlands, employed by the local or regional government, or the regional public health service. The inclusion criteria, developed to study the health effects of commuters' exposure to air pollution, were being of age between 18 and 56 years and non-smoking. Exclusion criteria were suffering from chronic obstructive pulmonary disease or asthmatic symptoms, using asthma medication, and being exposed at work to fumes or dust, to avoid confounding of exposures other than the studied air pollution exposure.

The measurements of the TRAVEL study were done between June 2007 and June 2008. During the commute by bus, car and bicycle, the volunteers were wearing heart rate monitors. Each volunteer participated at most 12 times. Each volunteer travelled by all transport modes. The commuting trips had a duration of two hours, from approximately 8 am to 10 am. During the car rides the volunteers were passengers, the car was driven by one of the researchers. Heart rates were recorded using Polar RS400 heart rate monitors (Polar Electro, Kempele, Finland). Heart rates were recorded per second. On the first six days of in total 47 days heart rates were recorded each five seconds, erroneously. We checked all graphs of the heart rate during commuting for abnormal patterns. For further analyses we excluded the parts where the heart rate was 'incorrect', 'incorrect' defined as a heart rate remaining exactly constant for 30 time points or longer. In addition we excluded the complete trip if the heart rate data were missing or 'incorrect' for more than 20% of the total travel time.

### Bicycle ergometer study

Minute ventilation levels of the commuters were estimated using heart rate measurements, because direct measurement of minute ventilation was not possible as it would influence inhalation of air pollutants. The submaximal tests to establish the relation between heart rate and minute ventilation were performed on bicycle ergometers, in June and December 2007 and in January 2008. The heart rate was recorded every second, using the same heart rate monitors as during the commute. The minute ventilation, breathing frequency and tidal volume were measured using a pneumotachometer (Jaeger, Viasys Healthcare, Hoechberg, Germany).

After a five to 10 minutes rest, the volunteer was positioned on the bicycle ergometer, wearing a facial mask to measure nose and mouth inhalation simultaneously. Heart rate and minute ventilation were measured at increasing cycling intensity, with measuring periods of one minute. The test started at rest, measuring minute ventilation and heart rate while the volunteer was seated on the ergometer, but not cycling. In the following test minutes the volunteer cycled at increasing speed/power, to measure at increasing heart rates. After cycling about a minute at the next level, when the heart rate was more or less stabilized, the heart rate and ventilation rate were measured again. This was continued until approximately 80% of the maximum heart rate had been reached. To calculate the maximum heart rate, the simple equations of 220 minus age (in years) for men, and 230 minus age for women were used. We did not measure at heart rates above 80% of the maximum, because higher heart rates were not expected to occur during the trips in the TRAVEL study and the relation between heart rate and minute ventilation may be different at higher heart rates. Per person the heart rate and minute ventilation was measured during 10 to 12 separate minutes.

We analyzed the series of minute-average heart rate and minute ventilation per person. Regression equations between heart rate and minute ventilation were calculated using the natural log transformation of minute ventilation, following the curvilinear relation between heart rate and ventilation used in previous studies [11-14] The effect of age, gender, height and body mass index on individual slopes and intercepts was analyzed using mixed models to take into account the effect of repeated measurements of heart rate and minute ventilation per person. For all analyses we used SAS 9.1 (SAS Institute Inc., Cary, NC, USA).

## Results

### Study population

In total 34 volunteers were recruited for the TRAVEL study. Table 1 shows the descriptive statistics of the population. The average body mass index (BMI) is similar to the average Dutch body mass index. Four people reported shortness of breath during exercise in the past 12 months, but not during rest, therefore they were not excluded from the study. The majority of the people did not consist of highly trained people. In the Netherlands most people will be able to cycle for two hours on a slightly less than normal speed, as the vast majority of the Dutch cycle regularly.

**Table 1:** Descriptive characteristics of the subjects (N = 34)

	Mean (min-max)
Age (yr)	42.0 (23-55)

BMI (kg/m^2^)	24.9 (19.0-30.8)

Gender	24 Male (71%)
	10 Female (29%)

Ex-smokers	10 (29%)

Education	5 Secondary school (15%)
	5 Vocational training (15%)
	24 College/university (71%)

Shortness of breath during exercise	4 (12%)

Nasal allergy (incl. hay fever)	5 (15%)

### Relation heart rate and minute ventilation

**Figure 1:**
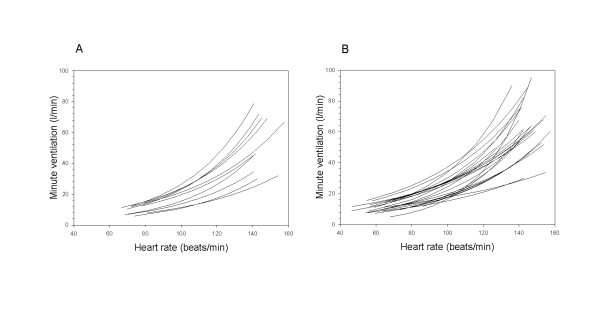
**Fitted regression lines of heart rate (beats per minute) and minute ventilation (litre per minute) for (A) 10 women and (B) 24 men**.

**Figure 2:**
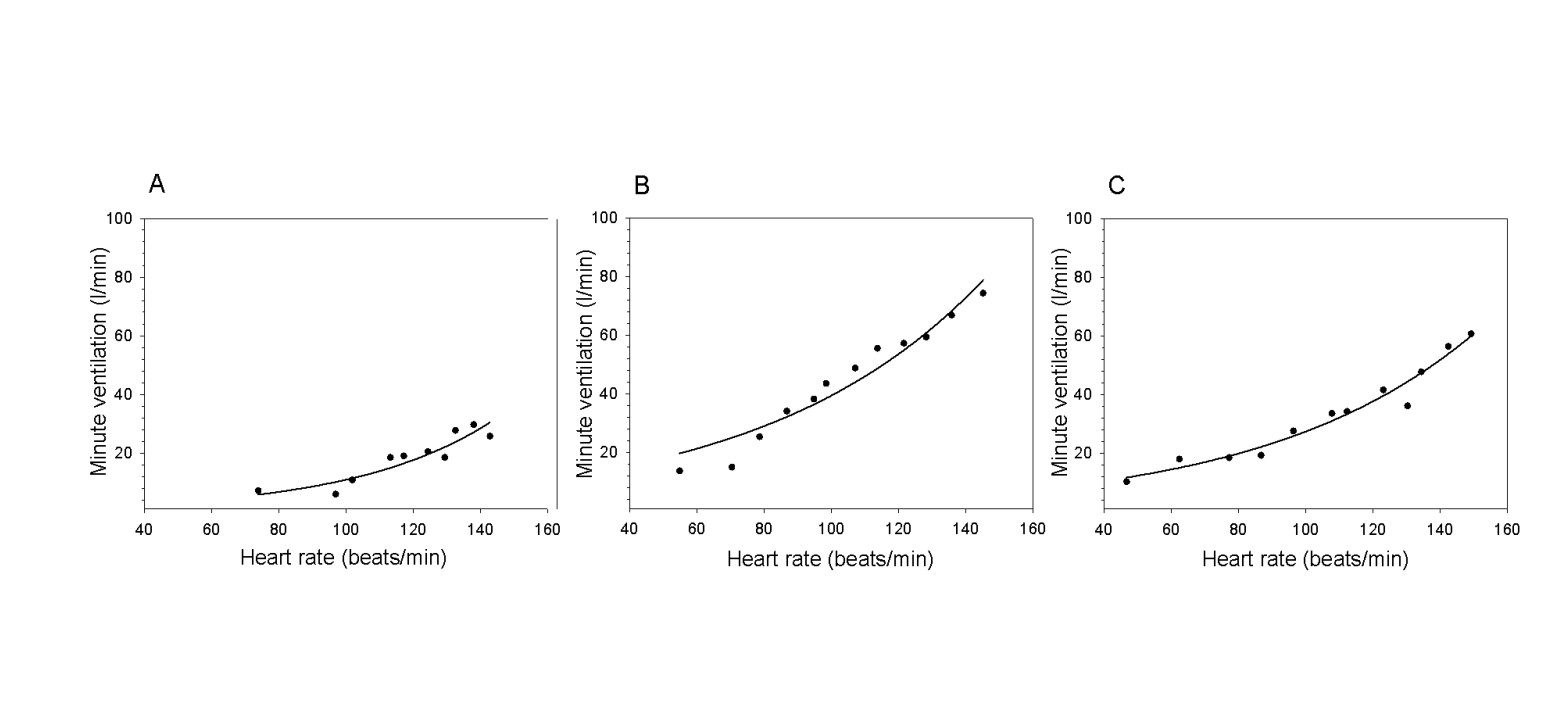
**Examples of fitted regression between heart rate and minute ventilation for (A) a female participant, R^2 ^of regression is 0.83 (B) a male participant, R^2 ^of regression is 0.92 (C) a male participant, R^2 ^of regression is 0.96**.

Regression equations between heart rate and natural log transformed minute ventilation were calculated for all 34 participants. The regression lines for all individuals are presented in figure 1A (women) and figure 1B (men). In table 2 the distribution of the regression coefficients for men, women and the total group are presented. The correlation between heart rate and minute ventilation is high, the mean R^2 ^is 0.90, suggesting that heart rate is a good predictor of minute ventilation. To illustrate the relationship between heart rate and minute ventilation, figure 2 shows scatter plots and fitted regression lines for three individuals. Examples are chosen as 10, 50 and 90 percentiles of all 34 R^2 ^values. We prepared plots for all individuals. Using linear fit of minute ventilation on heart rate without log-transformation gave similar fit, however, it resulted in negative minute ventilation predictions at low heart rates.

**Table 2:** Relationship between minute ventilation and heart rate during bicycle ergometer tests

	Intercept^#^		Slope^#^		R^2^	
	**Mean (SD)**	**Range**	**Mean (SD)**	**Range**	**Mean (SD)**	**Range**

All (n = 34)	0.89 (0.60)	-0.97-1.69	0.022 (0.005)	0.012-0.038	0.90 (0.07)	0.62-0.97

Men (n = 24)	1.03 (0.63)	-0.97-1.69	0.021 (0.005)	0.012-0.038	0.90 (0.07)	0.62-0.97

Women (n = 10)	0.57 (0.36)	-0.01-1.07	0.023 (0.003)	0.019-0.027	0.89 (0.06)	0.80-0.96

^#^Regression coefficients of the regression of natural log-transformed minute ventilation on heart rate.

There were modest differences in slopes and more substantial differences in intercepts between the individuals (table 2). The intercept is clearly lower in women than in men. Because of the curvilinear relation between heart rate and minute ventilation, the mean intercept of 1.03 of men with the mean slope of 0.022 results in a minute ventilation of 25 l/min at a heart rate of 100 bpm, while the mean intercept of women of 0.57 with equal slope and heart rate results in a minute ventilation of 16 l/min. Individual intercepts were negatively correlated with individual slopes and also negatively correlated with the individual mean heart rate during cycling. There was no correlation of intercepts or slopes with the increase in heart rate during cycling compared to car or bus riding. Mixed model analysis, using intercepts and heart rate as random effects, showed that there were significant differences in slopes and intercepts between subjects. 30% of the total variation in slope and in intercept was explained by between person variability.

In the mixed model analyses we used intercepts and heart rate as random effects. Age, height and body mass index were categorized in two groups, split at median level. There was an effect of gender on minute ventilation and heart rate, but no modifying effect of gender occurred. Neither analysis in the complete study population, nor separate analysis for male and female participants showed significant effect of age, height and body mass index on the relation between the natural logarithmic value of minute ventilation and heart rate, and no main effects of age, height or body mass index was found.

### Heart rate and minute ventilation during commuting

In total there were 357 commuting trips. We obtained 323 (90%) observations for the heart rate recordings during commuting. The missing 34 cases were due to heart rate monitors failures or volunteers forgetting to turn the heart rate monitors on. On some days the monitors made incorrect measurements of the heart rate, especially during the bus and car trips when people where not physically active so the dry skin did not make good contact with the heart rate monitor waistband. We excluded incorrect data from 38 trips. Five additional trips were excluded because the heart rate graphs showed too many irregularities. The final data set thus contained 280 of 357 trips (78%): 93 of 118 trips by car (79%), 80 of 113 trips by bus (71%) and 107 of 126 trips by bicycle (85%).

Using the individual relation between heart rate and minute ventilation derived from the bicycle ergometer tests, we calculated the minute ventilation of all individuals during all commuting trips (table 3). In addition, we calculated the ratio in minute ventilation between commuting by bicycle, bus and car for all participants (table 4). On average the minute ventilation on a bicycle in the study was 2.1 times higher than in a car, and 2.0 times higher than in the bus. These ratios varied considerably between the test persons. For women the difference in minute ventilation between cycling and car or bus riding was larger than for men. Minute ventilation values of car and bus passengers were similar. Leaving the individual with the lowest fit for heart rate and minute ventilation (R^2 ^= 0.62) out of the analysis did not change the results.

**Table 3:** Measured heart rate and estimated minute ventilation during commuting by bicycle, car and bus (n = 34)

	Heart rate^#^	Minute ventilation^$^
*All (n = 34)*		

Bicycle (n = 33)	100 (67 - 148)	23.5 (11.6 - 47.7)

Car (n = 33)	70 (52 - 99)	11.8 (5.1 - 20.9)

Bus (n = 32)	73 (52 - 95)	12.7 (5.4 - 19.5)

*Men (n = 24)*		

Bicycle (n = 24)	94 (67 - 122)	22.0 (11.6 - 29.5)

Car (n = 23)	66 (52 - 88)	11.9 (5.1 - 17.4)

Bus (n = 22)	70 (52 - 93)	13.1 (5.4 - 18.9)

*Women (n = 10)*		

Bicycle (n = 9)	116 (92 - 148)	27.6 (11.7 - 47.7)

Car (n = 10)	78 (70 - 99)	11.6 (6.8 - 20.9)

Bus (n = 10)	79 (70 - 95)	11.7 (7.0 - 19.5)

^#^Mean (min-max) of individual mean heart rate per commuting mode

^$^Mean (min-max) of individual mean minute ventilation per commuting mode

**Table 4:** Ratio of minute ventilation of commuting by bicycle, bus and car

	N	Mean	SD	Min	Max
*All (n = 34)*					

Ratio bicycle: bus	31	1.99	0.78	1.31	5.15

Ratio bicycle: car	32	2.09	0.77	1.34	5.30

Ratio bus: car	31	1.05	0.11	0.92	1.33

*Men (n = 24)*					

Ratio bicycle: bus	22	1.76	0.40	1.32	2.98

Ratio bicycle: car	23	1.88	0.38	1.34	3.16

Ratio bus: car	21	1.06	0.11	0.93	1.33

*Women (n = 10)*					

Ratio bicycle: bus	9	2.55	1.17	1.38	5.14

Ratio bicycle: car	9	2.61	1.20	1.58	5.30

Ratio bus: car	10	1.02	0.09	0.93	1.18

### Individual versus average regression of heart rate and minute ventilation

**Figure 3:**
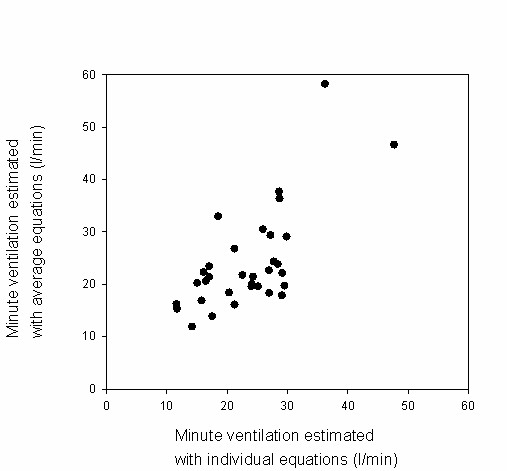
**Minute ventilation estimated by individual versus population average model**. On the x-axis minute ventilation is calculated using individual regression coefficients. On the y-axis minute ventilation is calculated using the mean regression coefficients calculated for all participants together, stratified by gender. Mean heart rates occurring during the cycling trips are used.

**Figure 4:**
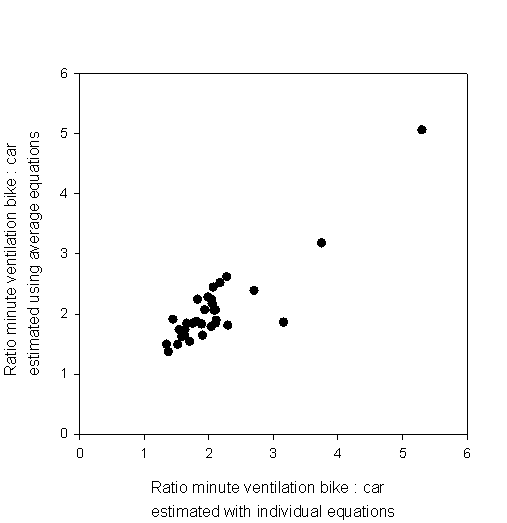
**Ratio of minute ventilation of cyclists compared to car passengers, calculated using individual versus population average model**. On the x-axis the ratio is based on minute ventilation levels of cyclists and car passengers, where minute ventilation levels are calculated using individual regression coefficients. On the y-axis the minute ventilation levels are calculated using the mean regression coefficients calculated for all participants together, stratified by gender. Mean heart rates occurring during the cycling and car trips are used.

In large studies it could be efficient to establish the relation between heart rate and minute ventilation for a sample of the total group, and to apply the mean regression coefficients to the total group. To illustrate the use of average coefficients compared to using individual regression coefficients, we calculated minute ventilation values using the mean slopes and intercepts, and using the mean heart rates of the participants during the bicycle rides, see table 5. In figure 3 these values have been plotted against the minute ventilation values calculated with the individual slopes and intercepts. The average difference between minute ventilation calculated in these two ways is small, but the figure shows that using the mean regression coefficients may lead to substantial differences in estimation of minute ventilation levels at individual level. The mean ratios of minute ventilation of cyclists compared to car passengers for both men and women are in line with the ratios calculated using personal coefficients. In figure 4 these ratios have been plotted against each other, showing that at individual level the use of mean coefficients may lead to large differences in ratios.

**Table 5:** Comparison of estimated minute ventilation using individual or group mean equations

	Minute ventilation cyclists		Ratio minute ventilation bike: car	
**Study/gender**	**Mean minute ventilation**	**Mean difference (l/min) (SD)^#^**	**Mean ratio**	**Mean difference (SD)^#^**

*TRAVEL: individual*^$^				

Men	22.0	NA	1.88	NA.

Women	27.6	NA	2.61	NA

*TRAVEL: mean*^†^				

Men	20.4	-1.7 (5.6)	1.87	-0.01 (0.36)

Women	34.4	6.8 (10.1)	2.57	-0.04 (0.32)

*Samet *[12]				

Men	26.9	4.9 (6.7)	1.92	0.03 (0.37)

Women	29.6	2.0 (9.5)	2.43	-0.18 (0.37)

*Colucci *[13]				

Men	10.0	-12 (4.8)	1.60	-0.29 (0.34)

^#^Mean difference with individual calculations (SD of difference)

^$^Using individual equations from this study

^†^Using group mean equations from this study

The average regression coefficients of heart rate and natural log transformed minute ventilation calculated in two other studies [12,13] are presented in table 6. We calculated the difference in estimated minute ventilation between using the average coefficients of Samet [12] and Colucci [13] and using our personal regression coefficients, making use of the individual mean heart rates occurring during the bicycle rides in the TRAVEL study. The use of the regression coefficients from Samet for men and women resulted in differences of 4.9 l/min (22%) and 2.0 l/min (7%), and using the equation of Colucci for men resulted in a difference of 12 l/min (51%), see table 5. We also calculated the ratio in minute ventilation of cyclists compared to car passengers, using the intercepts and slopes of Samet and Colucci, and making use of heart rates as occurred during the bicycle and car rides. The mean ratio of minute ventilation of cyclists compared to car passengers differs just slightly when using the equation of Samet for men. Using the equation of Samet for women and of Colucci (only available for men) underestimates the ratio, see table 5.

**Table 6:** Regression coefficients relation heart rate and natural log of minute ventilation compared to previous findings

Study/gender	Intercept (SD)	Slope (SD)
*TRAVEL, 2008*		

All (n = 34)	0.89 (0.60)	0.022 (0.005)

Men (n = 24)	1.03 (0.63)	0.021 (0.005)

Women (n = 10)	0.57 (0.36)	0.023 (0.003)

		

*Samet, 1993 *[12]		

Men (n = 15)	1.15 (0.60)	0.022 (0.004)

Women (n = 15)	0.72^#^(^$^)	0.022 (^$^)

		

*Colucci, 1982 *[13]		

Men	0.76 (^$^)	0.016 (^$^)

^#^Estimated from illustration in Samet, 1993 [12]

^$^Not reported

## Discussion

### Relation heart rate and minute ventilation

The bicycle ergometer tests showed that heart rate and minute ventilation are highly correlated, with substantial differences in regression equations between the individuals.

Minute ventilation during the bicycle rides were on average 2.1 times higher than in the car (individual range from 1.3 to 5.3) and 2.0 times higher than in the bus (individual range from 1.3 to 5.1). The ratio of minute ventilation during cycling compared to in bus or car was higher in women than in men.

Using overall equations for men and women instead of the individual coefficients resulted in good prediction of the mean minute ventilation levels of the population, but resulted in substantial differences in estimated minute ventilation on the individual level. Consequently, for studies on group level the use of overall equations, for instance obtained from a sample of the full study population, can be justified. However, when looking at individual level, the use of individual regression coefficients provides more precise data.

The correlation between heart rate and minute ventilation was high (average R^2 ^0.90) in the present study, as has also been shown before [12]. The kind of activity (upper-body activity or lower body activity) influences the relation between minute ventilation and heart rate [12]. In our study the use of bicycle ergometers is therefore appropriate for estimating the minute ventilation during cycling. Heart rate is not only influenced by exercise, but also by emotions, coffee, drugs, time of the day, temperature. These factors probably did not play an important role in this study.

The assumption of a log-linear relationship between heart rate and minute ventilation as used in this study, was based upon other publications [11-14]. The scatter plots (three of them presented in figure 2) and R^2 ^values of our 34 tests confirmed the good fit. Others have assumed a linear relation with one or two break points at the ventilatory compensation point (VCP) and/or the lactation threshold (LT) [15]. We did not have information about VCP or LT. Since we did not measure the full range of heart rate (up to maximum), we had limited possibilities to assess the shape of the relationship in our own data.

Other studies have used the oxygen uptake rate to estimate minute ventilation [16]. However, measuring oxygen uptake during commuting is difficult and would influence air pollution inhalation, therefore this method was not feasible in this study.

As shown in table 6, the average regression equations of heart rate and minute ventilation (natural log transformed) found in this study, were quite similar to that found by Samet [12] but differed somewhat from that of Colucci [13]. Individual slopes and intercepts differ considerably, as has also been reported before [12].

Applying the coefficients from Samet and overall equations from our own study instead of the individual coefficients, resulted in good prediction of the minute ventilation during cycling and of the mean ratio of minute ventilation of cyclists compared to car passengers. Using the coefficients from Colucci did not result in good predictions. However, details of this study, such as age of the test persons and number of persons, are missing, so we cannot estimate whether the study groups are truly comparable or not. The good results from the use of the average coefficients of Samet and our own average coefficients leads us to conclude that for studies on group level the use of overall equations can be justified. Large studies could estimate the relation between heart rate and minute ventilation for a sample of their population and apply it to the full population when only looking at mean group values.

The need for using individual slopes and intercepts instead of mean values in assessing minute ventilation levels at the individual level is underlined by figures 3 and 4. Minute ventilation levels during cycling and ratios of minute ventilation of cyclists to car passengers can differ widely when calculated using the mean regression coefficients instead of using the individual coefficients. We therefore conclude that for studies on individuals it is necessary to determine the individual relation between heart rate and minute ventilation, in agreement with previous studies [11,12,17].

### Minute ventilation levels during commuting

The volunteers did not drive the car in the study, but were seated in the back. The heart rates and relation between heart rate and minute ventilation in the car are therefore not much influenced by stress from traffic participation. The slightly higher heart rates in bus compared to car may be caused by more space in the bus to move around, though the volunteers kept seated. The US-EPA reported that minute ventilation levels of car drivers were only 10% higher compared to minute ventilation levels of car passengers [18]. The mean minute ventilation of car passengers in our study was 11.8 l/min, this is in line with the 12.3 l/min that has been measured in car drivers before [10].

The speed of the cyclists in the study was limited because of the limited speed of the technician cycling the bicycle loaded with heavy air monitoring equipment on partially hilly stretches. The average speed during the study was 12 km/h, while the average speed of cyclists in Dutch cities is estimated to be around 15 km/h, including stops while waiting for traffic lights. The differences in minute ventilation between cyclists and car and bus passengers are for most people therefore higher during cycling in everyday life than as determined in this study. In our study, minute ventilation of cyclists was on average 2.1 times higher than of car passengers, this is slightly lower than the factor 2.3 as has been calculated in a study where the speed of cyclists was not hampered [10]. In agreement, the mean minute ventilation of cyclists measured in the mentioned study [10] was 29.1 l/min while in our study the mean minute ventilation was 23.5 l/min. In a study on five young (20-32 years), male bicycle messengers, mean ventilation levels during cycling was 31 l/min, and mean heart rate during cycling was 107 bpm [14]. The bicycle messengers can be expected to cycle faster than the average cycling speed, so these data are also in line with our study, where mean minute ventilation of cyclists was 23.5 l/min and the mean heart rate was 100 bpm.

Intake of air pollutants is influenced by minute ventilation, but deposition of air pollutants is also influenced by the amount of nasal and oral breathing and by depth of inhalation. More oral breathing and deeper inhalation will occur during exercise, both leading to higher deposition of pollutants. In a study by Daigle et al [19] a 3.3-fold increase in minute ventilation led to a more than 4.5-fold increase in total ultrafine particle deposition.

In the present study we have not been able to measure oral and nasal breathing separately, nor have we measured the depth of inhalation. The ratios of minute ventilation of cyclists compared to car and bus passengers as calculated in the present study, are for those reasons likely to underestimate the true differences in deposition of air pollution inhaled by cyclists compared to car and bus passengers.

The inhaled dose of air pollutants of different groups of commuters is influenced by minute ventilation. We have shown that minute ventilation levels of cyclists are more than two times higher than commuters using a car or public transport. The increased minute ventilation of cyclists and other physically active commuters should be taken into account when comparing inhalation of air pollutants between different groups of commuters.

## Conclusion

This study demonstrates that the prediction of minute ventilation by measuring heart rates is a practical method for studies estimating inhaled of air pollution doses.

Individual regression equations are superior to overall equations in estimating minute ventilation levels using heart rate values. Finally, the minute ventilation of cyclists was on average 2.1 times higher than of car passengers and 2.0 times higher than of bus passengers.

Our study adds to the small database of minute ventilation during commuting. Our study strengthens the idea that average equations from our study or equations published before [11,12] can be used to estimate minute ventilation on population level in other, similar populations. The difference in minute ventilation between cyclists and car drivers/passengers so far had only been studied once before [10]. This study has not been published and only performed measurements in nine young adults. The findings of our study are in line with these findings and therefore give more certainty about the use of a factor two for differences in minute ventilation between commuting by bicycle or car or bus.

The study illustrates the importance of inclusion of minute ventilation data in comparing air pollution doses between different modes of transport.

## Abbreviations

TRAVEL: Transport Related Air Pollution, Variance in commuting, Exposure and Lung function; VCP: ventilatory compensation point; LT: lactation threshold; BMI: body mass index; SD: standard deviation.

## Competing interests

The authors declare that they have no competing interests.

## Authors' contributions

MZ participated in the design of the study, carried out the fieldwork, and drafted the manuscript. GH participated in the design of the study, assisted in statistical analyses and helped to draft the manuscript. PvdH and BB participated in coordination of the study and helped to draft the manuscript. All authors read and approved the manuscript.
